# Supplementary material for: A novel lossless encoding algorithm for data compression–genomics data as an exemplar
Source: Front Bioinform. 2025 Jan 23;4:1489704. doi: 10.3389/fbinf.2024.1489704 (PMC11799261; doi:10.3389/fbinf.2024.1489704)
Supplement: Supplementary file 4 [file DataSheet1.pdf]

Supplementary Materials

A Novel Lossless Encoding Algorithm for Data Compression - Genomics Data as an Exemplar

Anas Al-okaily[a], Abdelghani Tbakhi[b]

[a]Department of Cell Therapy and Applied Genomics, King Hussein Cancer Center, Amman, Jordan

[b]Department of Pathology and Molecular Medicine, McMaster University, Ontario, Canada

Contents

Material 1
Methods 1
Further Analysis and Investigations of the Proposed Algorithm 9
Table S6 10
Table S7 10
Table S8 10
Table S9 10
Table S10 10
Table S11 10
References 10

Material

In order to benchmark OST-DNA and the common compression tools, we used the tools provided in Table S1 with their descriptions. In addition, we used the genomes in Tables S2 and S3.

Methods

As discussed in the main text, all newlines, header lines, bases that are not A, C, G, T, or N were removed from the tested genomes to obtain a one-line sequence format for the each genome. The python script that was used for this purpose is stated below.

```
import sys
import os
import fileinput

fasta_file = sys.argv[1]
out_file =  open(os.path.basename(fasta_file).rsplit(".",1)[0] + ".one_line", 'wa')

# get all variants from each file without intersection
for line in fileinput.input(fasta_file):
    if line[0] == ">":
        pass
    else:
        for c in line:
            if c in ["A", "C", "G", "T", "N"]:
                out_file.write(c)
```

The size of each genomes before and after applying the python script is stated in Table S4.

Table S5 stated the commands used to compress one-line genomes for each tool. The same command which was used for bcm, brotli, bsc, bzip2, lrzip, lzip, and xz was also used to compress the bins in OST-DNA-bcm, OST-DNA-brotli, OST-DNA-bsc, OST-DNA-bzip2, OST-DNA-lrzip, OST-DNA-lzip, and OST-DNA-xz; respectively.

For unknown reason and given that the latest version of lrzip is v0.631, none of the versions later than v0.50 worked properly for the proposed algorithm, namely, in the step of compressing bins. So, v0.50 the version was used in this work.

The coding language which was used to implement OST-DNA versions is python 2.7.

The operating system and machine used for running and testing is as follows.

$ cat /etc/os-release

```
NAME="CentOS Linux"
VERSION="7 (Core)"
ID="centos"
ID_LIKE="rhel fedora"
VERSION_ID="7"
PRETTY_NAME="CentOS Linux 7 (Core)"
ANSI_COLOR="0;31"
CPE_NAME="cpe:/o:centos:centos:7"
HOME_URL="https://www.centos.org/"
BUG_REPORT_URL="https://bugs.centos.org/"
CENTOS_MANTISBT_PROJECT="CentOS-7"
CENTOS_MANTISBT_PROJECT_VERSION="7"
REDHAT_SUPPORT_PRODUCT="centos"
REDHAT_SUPPORT_PRODUCT_VERSION="7"
```

$ lscpu

```
Architecture:          x86_64
CPU op-mode(s):        32-bit, 64-bit
Byte Order:            Little Endian
CPU(s):                64
On-line CPU(s) list:   0-63
Thread(s) per core:    2
Core(s) per socket:    16
Socket(s):             2
NUMA node(s):          2
Vendor ID:             GenuineIntel
CPU family:            6
Model:                 79
Model name:            Intel(R) Xeon(R) CPU E5-2683 v4 @ 2.10GHz
```

Stepping: 1
CPU MHz: 1202.142
CPU max MHz: 3000.0000
CPU min MHz: 1200.0000
BogoMIPS: 4199.62
Virtualization: VT-x
L1d cache: 32K
L1i cache: 32K
L2 cache: 256K
L3 cache: 40960K

**Tool**	**Algorithm**	**Version**	**Year**	**Source Code**
bcm	Burrows-Wheeler transform	v1.51	2020	https://sourceforge.net/projects/bcm/
blzpack	brieflz library (https://github.com/jibsen/brieflz) which implements LZ style compression	v1.3.0	2020	https://github.com/jibsen/brieflz/tree/master/example
brotli	Google tool uses LZ77, Huffman encoding, and context modelling	v1.0.7	2018	https://github.com/google/brotli
bsc	Block sorting	v3.1.0	2016	https://github.com/IlyaGrebnov/libbsc
bzip2	Burrows-Wheeler transform	v1.0.8	2019	http://www.bzip.org/
cmix	Model prediction and Context mixing	v18	2019	https://github.com/byronknoll/cmix
compress	LZW algorithm	v4.2.4	2011	https://github.com/vapier/ncompress
freeze	LZSS and Huffman encoding	v2.5.0	1993	https://centos.pkgs.org/7/epel-x86_64/freeze-2.5.0-16.el7.x86_64.rpm.html
gzip	LZSS and Huffman coding	v1.10	2018	https://ftp.gnu.org/gnu/gzip/
hook	Dynamic Markov encoding	v0.8e	2007	http://cs.fit.edu/~mmahoney/compression/hook.zip
Huffman-codec	Huffman encoding	v1.0	2020	https://github.com/Bestoa/huffman-codec
lizard	LZ77 and Huffman encoding	v1.0	2019	https://github.com/inikep/lizard
lrzip	Extension of rzip but replacing bzip2 by LZMA, LZO, or no second-stage	v0.50	2010	http://ck.kolivas.org/apps/lrzip/
lz4	LZ77 family of byte-oriented compression schemes	v1.9.2	2019	https://github.com/lz4/lz4
lzb	Hexadecimal and base64 encoding/decoding	v1.0	2019	https://github.com/faragon/lzb
lzfse	Apple tool that applies LZ style compression using finite state entropy	v1.0	2017	https://developer.apple.com/documentation/comp

				ression/algorithm/lzfse
lzip	LZMA	v1.19	2017	https://www.nongnu.org/lzip/
lzop	LZO	v1.04	2017	https://www.lzop.org/
lzturbo	LZ77	v1.2	2014	https://sites.google.com/site/powturbo/
Nakamichi	LZSS	v1.0	2020	http://www.sanmayce.com/Nakamichi/
ppm	Prediction by partial matching	v1.0	2020	https://github.com/rene-puschinger/ppm
qzip	Quicklz (https://github.com/robottwo/quicklz) library which is based on LZRW	v0.2	2011	https://github.com/robottwo/quicklz
rans static	Arithmetic encoding	v1.0	2016	https://github.com/jkbonfield/rans_static
rzip	Encodes large chunks of duplicated data then uses bzip2	v1.0.6	2010	https://rzip.samba.org/
snzip	Snappy (https://github.com/google/snappy) which is a Google library based on ideas from LZ77	v1.0.4	2016	https://github.com/kubo/snzip
srank	Move-to-front encoding (symbol ranking)	v1.0	1997	https://www.cs.auckland.ac.nz/~peter-f/FTPfiles/srank.c
xz	LZ77 with huge dictionary sizes and range encoding	v5.2.2	2015	https://tukaani.org/xz/format.html
zlib	LZ77	v1.2.11	2017	https://github.com/madler/zlib
zip	LZ77	v3.0	2008	https://centos.pkgs.org/7/centos-x86_64/zip-3.0-11.el7.x86_64.rpm.html
zpipe	ZLib implementation of gzip and deflate	v1.0	2017	https://github.com/skyformat99/zpipe
zstd	Facebook tool uses LZ77, finite-state-entropy, and Huffman encoding	v1.4.5	2020	https://github.com/facebook/zstd

Table S1: Most common general-purpose compression tools. Latest version for each tool was installed except for lrzip (the reason for this is provided in Supplementary Materials and Methods).

**Category**	**Organism**	**Accession**	**Size (byte)**
Virus	Gordoniaphage GAL1 [1]	GCF 001884535.1	50,654
Bacteria	WS1 bacterium JGI 0000059-K21 [2]	GCA 000398605.1	521,951
Protist	Astrammina rara [2]	GCA 000211355.2	1,712,167
Fungus	Nosema ceranae [2]	GCA 000988165.1	5,809,207
Protist	Cryptosporidium parvumIowa II [2]	GCA 000165345.1	9,216,802
Protist	Spironucleus salmonicida [2]	GCA 000497125.1	13,142,503
Protist	Tieghemostelium lacteum [2]	GCA 001606155.1	23,672,980

Fungus	Fusarium graminearumPH-1 [1]	GCF 000240135.3	36,915,673
Protist	Salpingoeca rosetta [2]	GCA 000188695.1	56,150,373
Algae	Chondrus crispus [2]	GCA 000350225.2	106,387,446
Algae	Kappaphycus alvarezii [2]	GCA 002205965.2	341,012,624
Animal	Strongylocentrotus purpuratus [1]	GCF 000002235.4	1,007,867,539
Plant	Picea abies [2]	GCA 900067695.1	13,409,043,938

Table S2. Genome sequence datasets

**Dataset**	**No. of sequences**	**Size (byte)**	**Source**	**Date**
Mitochondrion *[1]*	9,402	245,282,526	RefSeq ftp: ftp://ftp.ncbi.nlm.nih.gov/refseq/release/mitochondrion/mitochondrion.1.1.genomic.fna.gz ftp://ftp.ncbi.nlm.nih.gov/refseq/release/mitochondrion/mitochondrion.2.1.genomic.fna.gz	15 March 2019
NCBI Virus Complete Nucleotide Human *[3]*	36,745	481,767,318	NCBI Virus: https://www.ncbi.nlm.nih.gov/labs/virus/vssi/	11 May 2020
Influenza [4]	700,001	1,215,166,594	Influenza Virus Database: ftp://ftp.ncbi.nih.gov/genomes/INFLUENZA/influenza.fna.gz	27 April 2019
Helicobacter [1]	108,292	2,756,472,239	NCBI Assembly: https://www.ncbi.nlm.nih.gov/assembly	24 April 2019

Table S3. Other DNA datasets

**Genome**	**Size of genome in fasta format (byte)**	**Size after applying scripts (one-line format) (byte)**
**DNA-Genome-Picea-abies-GCA_900067695.1-2016-11-09**	13,409,043,938	11,960,690,255

**DNA-Helicobacter-2019-04-24**	2,756,472,239	2,711,601,413
**DNA-Influenza-2019-04-27**	1,215,166,594	1,106,180,040
**DNA-Genome-Strongylocentrotus-purpuratus-GCF_000002235.4-2015-03-10**	1,007,867,539	659,082,267
**DNA-NCBI-Virus-Complete-Nucleotide-Human-2020-05-11**	481,767,318	470,877,038
**DNA-Genome-Kappaphycus-alvarezii-GCA_002205965.2-2018-03-09**	341,012,624	249,541,865
**DNA-Mitochondrion-2019-03-15**	245,282,526	241,606,731
**DNA-Genome-Chondrus-crispus-GCA_000350225.2-2013-05-22**	106,387,446	77,592,685
**DNA-Genome-Salpingoeca-rosetta-GCA_000188695.1-2011-02-10**	56,150,373	37,299,580
**DNA-Genome-Fusarium-graminearum-PH-1-GCF_000240135.3-2008-11-21**	36,915,673	34,133,023
**DNA-Genome-Tieghemostelium-lacteum-GCA_001606155.1-2016-04-04**	23,672,980	17,671,254
**DNA-Genome-Spironucleus-salmonicida-GCA_000497125.1-2013-11-19**	13,142,503	9,934,367
**DNA-Genome-Cryptosporidium-parvum-Iowa-II-GCA_000165345.1-2007-02-26**	9,216,802	7,012,512
**DNA-Genome-Nosema-ceranae-GCA_000988165.1-2015-05-05**	5,809,207	3,541,094
**DNA-Genome-Astrammina-rara-GCA_000211355.2-2011-04-27**	1,712,167	1,362,918
**DNA-Genome-WS1-bacterium-JGI-0000059-K21-GCA_000398605.1-2013-05-16**	521,951	509,551
**DNA-Genome-Gordonia-phage-GAL1-GCF_001884535.1-2016-11-15**	50,654	49,979
**Total size in Megabytes**	**18,797.10**	**16,773.88**

Table S4. Size of each genome before and after applying the script

Tool	Compression command	Decompression command
**bcm**	bcm $genome_name	bcm -d -f $genome_name.bcm
**blzpack**	blzpack $genome_file $genome_name.blz	blzpack $genome_name.blz $genome_file
**brotli**	brotli $genome_file -c > $genome_name.br	brotli -d $genome_name.br -c > $genome_file

**bsc**	bsc e $genome_file $genome_name.bsc -t -T	bsc d $genome_name.bsc $genome_file
**bzip2**	bzip2 -k -c $genome_file > $genome_name.bz2	bunzip2 $genome_name.bz2
**cmix**	cmix -c $genome_file ./$genome_name.cmix	cmix -d ./$genome_name.cmix ./$genome_file
**compress**	compress $genome_file -c > $genome_name.Z	compress -d $genome_name.Z -c > $genome_file
**freeze**	freeze -c $genome_file > $genome_name.F	freeze -d -c $genome_file > $genome_file
**gzip**	gzip $genome_file -c > $genome_name.gz	gunzip -d $genome_name.gz -c > $genome_file
**HE**	huffman -e $genome_name > $genome_name.hf	huffman -d $genome_name.hf > $genome_file
**hook**	hook08e c 256 2 1 255 $genome_file $genome_name.hk	hook08e d $genome_name.hk
**lizard**	lizard -f -c $genome_file > $genome_name.lzd	lizard -f -c -d $genome_name.lzd > $genome_file
**lrzip**	lrzip -q -N 1 -f $genome_file -o $genome_name.lrz	lrzip -d -q -N 1 -f $genome_name.lrz -o $genome_file
**lz4**	lz4 $genome_file -c > $genome_name.lz4	lz4 -d $genome_name.lz4 -c > $genome_file
**lzb**	lzb < $genome_file > $genome_name.lzb	lzb -d < $genome_name.lzb > $genome_file
**lzfse**	lzfse -encode -i $genome_file > $genome_name.lzfse	lzfse -decode -i $genome_name.lzfse > $genome_file
**lzip**	lzip -c $genome_file > $genome_name.lz	lzip -d -c $genome_name.lz > $genome_file
**lzop**	lzop $genome_file -c > $genome_name.lzo	lzop -d $genome_name.lzo -c > $genome_file
**LzTurbo**	lzturbo -32 -o -f $genome_file > $genome_name.lzt	lzturbo -32 -o -f -d $genome_name.lzt > $genome_file
**nakamichi**	nakamichi $genome_file $genome_name.Nakamichi	nakamichi $genome_name.Nakamichi > $genome_file
**ppm**	ppm c $genome_file $genome_name.ppm	ppm d $genome_name.ppm $genome_file
**qzip**	qzip $genome_name	qunzip -d $genome_name.qz
**rans**	rANS_static -o1 $genome_file $genome_name.rans1	rANS_static -d $genome_name.rans1 $genome_file

**rzip**	rzip -k $genome_file -o $genome_name.rz	rzip -d -f $genome_name.rz
**snzip**	snzip -c $genome_file > $genome_name.sz	snzip -d -c $genome_name.sz > $genome_file
**srank**	srank $genome_file	srank $genome_name.sr
**xz**	xz -z -c $genome_file > $genome_name.xz	xz -d $genome_name.xz -c > $genome_file
**zip**	zip $genome_name.zip $genome_file	unzip $genome_name.zip
**zlib**	openssl zlib -e -in $genome_file > $genome_name.zlib	openssl zlib -d -in $genome_name.zlib > $genome_file
**zpipe**	zpipe < $genome_file > $genome_name.z	zpipe -d < $genome_name.z > $genome_file
**zstd**	zstd -f $genome_file -o $genome_name.zst	zstd -d -f $genome_name.zst

Table S5. Commands used to compress files for each tool

Further Analysis and Investigations of the Proposed Algorithm

Data binning is the underlying process in the proposed algorithm. Each sequence of length *w* (or of variable length) in *S* must be labeled and assigned to a bin with the corresponding label. Sequences in the same bin, which are assumed to be similar to each other, are then encoded and compressed together. Bins with different labels can be encoded and compressed using either a single encoding or a specific algorithm. The process used to compress a bin can be determined by the label and/or content of the bin.

This approach raises several questions that need to be addressed beyond this study. Given a set of concatenated or non-concatenated sequences sharing similar characteristics (e.g., the same Huffman tree), can an efficient or optimal encoding/compression solution be identified? What is the optimal classification/labeling method for bins? What is the optimal number of bins to be used? Is it better to use a window length that is fixed, variable, or dependent on the text/genome length?

Data binning has been used to solve compression problems for sequence reads, but not for genomic sequences or textual data. Assembltrie [5], BEETL [6], BdBG [7] FaStore [8], HARC [9], Mince [10], Orcom [11], and SCALCE [12] apply data binning to the compression of short variable-length sequences. There are several differences between these techniques and OST. First, these algorithms are typically applied to short sequences (of a few hundred bases in length), unlike genome or textual data. Second, the sequencing of such reads is typically conducted at high coverage. Hence, redundancy often exists between the reads, which is a motivating factor for the application of a binning process. Third, none of these algorithms preserve the order of the inputted reads.

The OST algorithm is designed primarily for long sequences in which there is no assumption of high repetitiveness in the input, as not all genomes or textual data exhibit such patterns and are potentially random. A key step in OST is to encode and record the order of the subsequences by recording the order of labels in L to preserve the sub-sequence placement in the original input. This allows the data to be completely and exactly restored during the decompression process.

Table S6
Compression results using each tool in Table 1 for each one-line genome.
Table S7
Compression results using each OST-DNA version with each window-length and each label-length for each one-line genome.
Table S8
Compression results using each OST-DNA version with each window-length and each label-length for all one-line genomes cumulatively.
Table S9
Compression results using each OST-DNA version for each bin produced from each one-line genome, each window-length, and each label-length.
Table S10
Compression results using each OST-DNA version for each bin produced from same window-length and same label-length but from different genomes.
Table S11
Compression results using each OST-DNA version for each bin produced from same label-length and same genome but different window-lengths.

References

1. N. A. O'Leary, M. W. Wright, J. R. Brister, S. Ciufo, D. Haddad, R. McVeigh, B. Rajput, B. Robbertse, B. Smith- White, D. Ako-Adjei, et al. Reference sequence (refseq) database at ncbi: current status, taxonomic expansion, and functional annotation. Nucleic acids research, 44(D1): D733–D745, 2016.
2. K. Clark, I. Karsch-Mizrachi, D. J. Lipman, J. Ostell, and E. W. Sayers. Genbank. Nucleic acids research, 44(D1): D67–D72, 2016.
3. J. R. Brister, D. Ako-Adjei, Y. Bao, and O. Blinkova. Ncbi viral genomes resource. Nucleic acids research, 43(D1): D571–D577, 2015.
4. Y. Bao, P. Bolotov, D. Dernovoy, B. Kiryutin, L. Zaslavsky, T. Tatusova, J. Ostell, and D. Lipman. The influenza virus resource at the national center for biotechnology information. Journal of virology, 82(2):596–601, 2008.
5. A. A. Ginart, J. Hui, K. Zhu, I. Numanagi´c, T. A. Courtade, S. C. Sahinalp, and N. T. David. Optimal compressed representation of high throughput sequence data via light assembly. Nature communications, 9(1):1–9, 2018.

6. A. J. Cox, M. J. Bauer, T. Jakobi, and G. Rosone. Large- scale compression of genomic sequence databases with the burrows–wheeler transform. Bioinformatics, 28(11):1415– 1419, 2012.
7. R. Wang, J. Li, Y. Bai, T. Zang, and Y. Wang. Bdbg: a bucket- based method for compressing genome sequencing data with dynamic de bruijn graphs. PeerJ, 6:e5611, 2018.
8. L- . Roguski, I. Ochoa, M. Hernaez, and S. Deorowicz. Fastore: a space-saving solution for raw sequencing data. Bioinformatics, 34(16):2748–2756, 2018.
9. S. Chandak, K. Tatwawadi, and T. Weissman. Compression of genomic sequencing reads via hash-based reordering: algorithm and analysis. Bioinformatics, 34(4):558–567, 2018.
10. R. Patro and C. Kingsford. Data-dependent bucketing improves reference-free compression of sequencing reads. Bioinformatics, 31(17):2770–2777, 2015.
11. S. Grabowski, S. Deorowicz, and L- . Roguski. Disk-based compression of data from genome sequencing. Bioinformatics, 31(9):1389–1395, 2015.
12. F. Hach, I. Numanagi´c, C. Alkan, and S. C. Sahinalp. Scalce: boosting sequence compression algorithms using locally consistent encoding. Bioinformatics, 28(23):3051– 3057, 2012.
